# Seasonal Effects on Corneal Densitometry and Haze After Mitomycin C-Assisted Photorefractive Keratectomy

**DOI:** 10.3390/jcm15124584

**Published:** 2026-06-12

**Authors:** Halil Emre Özdemir, Muzaffer Talha Albayrak, Yusuf Koçluk

**Affiliations:** 1 Department of Ophthalmology, Büyük Anadolu Hospital, 41700 Darıca, Kocaeli, Türkiye; halilemre53@gmail.com; 2 Department of Ophthalmology, Kayseri State Hospital, 38010 Kocasinan, Kayseri, Türkiye; 3 Department of Ophthalmology, Medline Hospital, 01170 Çukurova, Adana, Türkiye; kocluk99@gmail.com

**Keywords:** photorefractive keratectomy, corneal haze, corneal densitometry, ultraviolet radiation, seasonal variation

## Abstract

**Purpose**: To investigate whether the season in which photorefractive keratectomy (PRK) with mitomycin C (MMC) is performed influences postoperative corneal transparency considering seasonal variations in ultraviolet (UV) exposure. **Methods**: This retrospective study included 100 eyes of 50 patients who underwent MMC-assisted PRK for myopic refractive error. Patients were divided into two groups based on the season of surgery: winter (November–April, low UV exposure) and summer (May–October, high UV exposure). All patients were evaluated preoperatively and at 12 months postoperatively. Corneal transparency was objectively assessed using Scheimpflug-based corneal densitometry obtained with the Pentacam system, focusing on the central (0–2 mm) and paracentral (2–6 mm) zones. Postoperative densitometry values were compared between seasonal groups. **Results**: Corneal densitometry values showed a significant reduction postoperatively compared with preoperative measurements in both central and paracentral zones. When comparing seasonal groups, no statistically significant differences were observed in postoperative densitometry values between eyes operated on during summer and winter months. No clinically detectable corneal haze was observed in any patient throughout the follow-up period. **Conclusions**: Surgical season did not significantly influence postoperative corneal densitometry or clinically detectable haze formation after MMC-assisted PRK for low-to-moderate myopia, suggesting that deferral during high-UV months may not be necessary with standard postoperative UV protection.

## 1. Introduction

The primary approaches to correcting refractive errors include spectacles, contact lenses, and refractive surgery [1,2,3]. When non-surgical options are insufficient or not preferred, surgical interventions such as photorefractive keratectomy (PRK) are frequently employed [3,4,5].

However, PRK is associated with several postoperative complications, including diffuse lamellar keratitis, corneal haze, optical aberrations, punctate epithelial keratopathy, infectious keratitis, corneal ectasia, and subepithelial hemorrhage. Among these, corneal haze is considered one of the most serious complications, as it can significantly degrade visual quality, be more challenging to manage, and in severe cases potentially lead to permanent vision loss. Ultraviolet (UV) radiation has been implicated as an important trigger for corneal haze formation after refractive surgery, and increased postoperative UV exposure may further elevate this risk [6,7,8,9,10]. Therefore, it was hypothesized that seasonal differences in ambient UV exposure may influence postoperative corneal transparency and haze development in patients undergoing MMC-assisted PRK.

Corneal haze is traditionally assessed by slit-lamp biomicroscopy, though this method is subjective and examiner-dependent. For objective evaluation, Scheimpflug-based tomography and confocal microscopy are employed. While confocal imaging is contact-based and less comfortable, Scheimpflug imaging offers a rapid, non-contact alternative, with densitometry values serving as reliable quantitative indicators of corneal clarity [11,12,13,14,15].

Although UV exposure has been implicated in post-PRK haze formation, it remains unclear whether seasonal variation in ambient UV exposure affects postoperative corneal transparency after MMC-assisted PRK. Therefore, this study evaluated postoperative corneal densitometry and clinical haze outcomes by comparing surgeries performed during high-UV months (May–October) with those performed during lower-UV months (November–April) under real-life clinical conditions.

## 2. Materials and Methods

This retrospective study reviewed the records of 100 eyes from 50 patients who underwent photorefractive keratectomy combined with Mitomycin C (PRK + MMC) (WaveLight EX500 Excimer Laser, Alcon Laboratories, Inc., Fort Worth, TX, USA) for myopic refractive error at the Cornea Unit of Adana City Training and Research Hospital between January 2018 and January 2020. In all cases, the same standardized laser protocol was applied; after epithelial removal, stromal ablation was performed using a standardized 6.5-mm optical zone. All consecutive patients who met the predefined inclusion and exclusion criteria were included in the study.

Data from these 100 eyes were recorded at two time points: preoperatively and at the final follow-up visit (12 months postoperatively). Demographic data including age and gender, preoperative and postoperative visual acuity (measured with the Snellen chart), anterior segment examination findings, and corneal tomography data (Pentacam, Oculus Optikgeräte GmbH, Wetzlar, Germany) were collected. Corneal densitometry values derived from the integrated densitometry module of the same device were also recorded.

Corneal Densitometry (CD): CD values are expressed in Gray Scale Units (GSU), ranging from 0 to 100, where 0 corresponds to complete transparency and 100 denotes completely opaque corneal tissue that blocks all light. The Pentacam densitometry module allows measurement of densitometry values across different regions and depths of the cornea within a 12 mm diameter zone. The cornea is divided into four concentric zones: 0–2 mm, 2–6 mm, 6–10 mm, and 10–12 mm. Additionally, densitometry values can be measured at three different corneal depths: the anterior layer (superficial 120 µm), the posterior layer (innermost 60 µm), and the central stroma located between these two layers. In this study, densitometry measurements were analyzed specifically in two zones: the central 0–2 mm zone (CD02) and the 2–6 mm paracentral zone (CD26). Clinical corneal haze was assessed by slit-lamp biomicroscopy using the Fantes 0–4 grading scale during postoperative follow-up visits, in addition to objective evaluation with Pentacam densitometry.

The study further investigated whether postoperative findings and complications differed according to the season in which surgery was performed. Corneal haze and densitometry values were analyzed in relation to expected postoperative ambient UV exposure. Patients who underwent surgery during the winter months (November–April), a period with limited sunlight exposure, were designated as Group 1. Those who had surgery in the summer months (May–October), when ambient UV exposure is high in the Mediterranean region where the study was conducted, were assigned to Group 2. This seasonal classification was based on published regional UV radiation data from the Adana region, which demonstrate substantially higher UV exposure between May and October and lower UV exposure between November and April. Preoperative and postoperative parameters were compared between these groups. To ensure that the seasonal UV exposure classification reflected the patients’ actual postoperative environmental conditions, only patients residing in the Adana region during the postoperative recovery period were included in the study. Patients who lived outside the Adana region or who were known to have spent the early postoperative period in another geographic region were excluded from the analysis.

Postoperatively, patients were advised to rest with their eyes closed immediately after the procedure. Pain control was provided with oral nonsteroidal anti-inflammatory drugs for 24–48 h when needed. All patients received the same standard topical treatment regimen, including topical moxifloxacin, dexamethasone, preservative-free artificial tears, and/or epithelializing lubricant drops four times daily. The bandage contact lens and topical antibiotic therapy were continued until complete epithelial healing was achieved. Topical corticosteroid treatment was continued for 1 month. Compliance with the postoperative medication protocol was assessed from follow-up records, and patients with documented noncompliance were excluded from the study. In addition, all patients received the same standard postoperative UV-protection recommendations routinely provided after refractive laser procedures. These included avoiding unnecessary direct sunlight exposure and wearing UV-protective sunglasses when outdoors during the early postoperative period, regardless of the season in which surgery was performed. The aim of the present study was not to quantify unrestricted individual UV exposure, but rather to evaluate, under real-life clinical conditions, whether the season of surgery was associated with different postoperative haze or corneal densitometry outcomes among patients who received routine postoperative UV-protection advice. However, individual adherence to these recommendations and the actual duration or intensity of outdoor UV exposure were not objectively recorded. The UV radiation values cited for the Adana region represent published regional seasonal averages rather than daily UV measurements linked to each individual surgery date during the study period. Therefore, seasonal grouping was used as a pragmatic surrogate for expected ambient UV exposure. Although a plot of daily UV radiation levels during the study period with annotated surgery dates would provide more granular exposure characterization, such daily local UV data linked to individual surgical dates were not available in this retrospective dataset.

Inclusion Criteria: Patients aged 18 years and older, phakic eyes, and a minimum follow-up period of 12 months.

Exclusion Criteria: Patients were excluded if they had any additional ocular disease, systemic disease, previous ocular surgery, incomplete preoperative or follow-up examination records, incomplete corneal topography data, noncompliance with the standard postoperative treatment protocol, or failure to attend the final follow-up visit despite attempts to contact them. In addition, patients residing outside the Adana region or those who spent the early postoperative recovery period in another geographic region were excluded to ensure that the seasonal UV exposure classification reflected the postoperative environmental conditions of the study population.

### Statistical Analysis

Statistical analyses were performed using IBM SPSS for Windows version 21.0 (IBM SPSS Inc., Chicago, IL, USA). The normality of the data distribution was assessed with the Kolmogorov–Smirnov test. Quantitative variables were presented as mean ± standard deviation (minimum–maximum). For the comparison of preoperative and postoperative corneal densitometry (CD) measurements, Generalized Estimating Equations (GEE) were employed. GEE models were used to account for the correlation between fellow eyes within the same patient, allowing both eyes to be included in the analysis while appropriately addressing their non-independence. Within the GEE framework, a linear model with an exchangeable working correlation matrix was used to evaluate the main effects of time and seasonal group, as well as their interaction. In the primary GEE models, corneal densitometry values were entered as the dependent variable, while time point, seasonal group, and the time-by-season interaction were included as fixed effects. Patient identity was used as the clustering variable to account for inter-eye correlation, and separate models were constructed for CD02 and CD26. Baseline refractive parameters, including preoperative spherical diopter, astigmatism, spherical equivalent, and ablation depth, were also compared between seasonal groups using separate GEE models. For other independent between-group comparisons of numerical variables, the independent samples *t*-test or Mann–Whitney U test was used, as appropriate. Qualitative variables were expressed as percentages and compared using the chi-square test. A *p*-value less than 0.05 was considered statistically significant.

## 3. Results

A total of 100 eyes from 50 patients who underwent PRK + MMC for refractive error correction were evaluated preoperatively and at the final follow-up. The mean follow-up duration was 14.40 ± 1.45 months (range: 12–18 months). The mean age of the patients was 24.52 ± 5.36 years (range: 20–54 years). Among these patients, 25 (50%) were female and 25 (50%) were male. Surgery was performed on the right eye in 50 cases (50%) and on the left eye in 50 cases (50%). Pre- and postoperative examination findings and collected data related to PRK + MMC are presented in Table 1.

### 3.1. Corneal Densitometry

CD values from different corneal zones were compared preoperatively and postoperatively, and the results are presented in Table 2. A statistically significant decrease was observed in both zones.

### 3.2. Comparative Analysis Between Groups

Group 1 consisted of 42 eyes, and Group 2 included 58 eyes. Comparative analyses were conducted between the groups. The mean age was 26.90 ± 9.15 years in Group 1 and 23.83 ± 3.29 years in Group 2, with no statistically significant difference observed (*p* = 0.298). Preoperative spherical diopter, astigmatism, spherical equivalent, and ablation depth were compared between the seasonal groups using GEE models to account for inter-eye correlation within the same patient. No statistically significant differences were observed between the groups for preoperative spherical diopter (*p* = 0.415), astigmatism (*p* = 0.491), spherical equivalent (*p* = 0.542), or ablation depth (*p* = 0.643), indicating comparable baseline refractive and treatment profiles. Therefore, these parameters were not included as additional covariates in the final densitometry models.

Preoperative and postoperative zone-specific corneal densitometry values, specifically CD02 and CD26, were analyzed and compared between the groups using GEE models to account for inter-eye correlation within the same patient. The models included time point, seasonal group, and the time-by-season interaction. There were no statistically significant differences in preoperative corneal densitometry values between the seasonal groups for either parameter (CD02, *p* = 0.168; CD26, *p* = 0.284), indicating the absence of a seasonal effect at baseline. In contrast, both CD02 and CD26 values showed a statistically significant reduction from preoperative to postoperative measurements within each group (all *p* < 0.001) (Table 3).

## 4. Discussion

Trokel et al. first performed PRK in 1983 using argon fluoride laser for corneal ablation [16]. Over the years, PRK has been widely accepted as a reliable procedure [17,18,19]. Our study’s refractive outcomes are also consistent with the existing literature, demonstrating a statistically significant refractive improvement following PRK. However, concerns such as the potential development of corneal haze, postoperative pain, and delayed stabilization of refraction and vision have led to the adjunctive use of MMC with PRK, as well as the exploration of alternative techniques including LASIK, transepithelial PRK (T-PRK), and SMILE [9,20,21,22].

Haze formation is a well-documented complication following refractive surgery, particularly PRK, with increased risk in cases involving high-degree corrections and postoperative exposure to UV radiation. To mitigate this risk, Mitomycin C (MMC) is frequently employed due to its proven efficacy in reducing postoperative stromal haze [23,24,25]. In a study by Almosa et al., 159 eyes from 80 patients undergoing PRK were evaluated for postoperative haze formation. The group that did not receive MMC showed some instances of haze, whereas no cases were observed in the MMC-treated group, supporting the potential effectiveness of MMC in reducing haze [23]. Similarly, Coelho and colleagues compared two concentrations of MMC 0.02% and 0.002% administered for 30 s in each group. The study included 130 patients and utilized a biomicroscopic haze grading scale from 0 to 4. At the one-year follow-up, the incidence of haze was 7.7% in the 0.02% group and 5.4% in the 0.002% group. However, the difference between the groups was not statistically significant, suggesting that lower concentrations of MMC may be similarly effective under certain conditions [24]. In parallel with previous literature suggesting that MMC may reduce the risk of postoperative haze, all eyes in the present study underwent PRK with intraoperative MMC application. Within this MMC-assisted PRK cohort, no clinically detectable postoperative haze was observed during follow-up; however, because a non-MMC control group was not included, the present study was not designed to determine the independent protective effect of MMC.

Corneal haze can develop in association with various conditions, including corneal degenerations, dystrophies, trauma, keratitis, and refractive surgical procedures. Traditionally assessed subjectively via slit-lamp biomicroscopy, haze can now also be evaluated quantitatively using corneal densitometry, thanks to advancements in corneal tomography. Densitometry measures the intensity of backscattered light from the cornea, offering objective data on corneal transparency [6,26,27,28]. Cennamo et al. compared densitometry changes in myopic eyes undergoing PRK with a control group. In their study, a statistically significant decrease in anterior corneal densitometry was observed at 12 months postoperatively compared to baseline, demonstrating that corneal transparency is maintained in the long term [29]. Consistent with the literature, a statistically significant reduction in corneal densitometry values was observed from baseline to the final follow-up in our study, suggesting that corneal transparency was maintained without densitometric evidence of increased haze over the long term. The postoperative decrease in densitometry may be partly explained by the removal of anterior stromal tissue during PRK, as the anterior corneal layers are known to contribute more prominently to backscattered light than deeper stromal layers. In addition, postoperative reductions in corneal thickness and total corneal volume after stromal ablation may also contribute to lower measured densitometry values. However, this interpretation should be considered cautiously, because PRK also induces epithelial remodeling and stromal wound-healing responses, both of which may influence postoperative corneal optical density. Therefore, the observed reduction in densitometry is likely multifactorial rather than attributable to a single mechanism.

This study was conducted in Adana, a city in the southern region of Turkey characterized by high annual sunlight intensity. Ground-based ultraviolet (UV) radiation measurements from the Adana region demonstrate a pronounced seasonal variation in surface UV exposure. UV radiation levels are substantially higher during the summer months, with peak mean daily values observed in July (0.96 MJ/m^2^), whereas markedly lower levels are recorded during the winter season, reaching minimum values in January (0.27 MJ/m^2^). Overall, summer UV exposure in this region is approximately three to four times greater than that observed in winter, highlighting a clear seasonal contrast in ultraviolet radiation intensity [30]. When interpreted in the context of the Global Solar UV Index classification, these regional findings support the characterization of the study region as a clinically relevant high-UV Mediterranean environment rather than an extreme global UV-exposure setting [31].

In the present study, 100 eyes from 50 patients who underwent PRK with intraoperative mitomycin C were stratified into two groups according to the season in which the procedure was performed. Group 1 comprised patients operated on during the winter months (November to April), whereas Group 2 included those who underwent surgery in the summer months (May to October). Based on the hypothesis outlined above, postoperative corneal densitometry values were assessed as objective indicators of corneal haze at the final follow-up using the Pentacam imaging system. Specifically, densitometry measurements from the central (CD02) and paracentral (CD26) corneal zones were compared between the two groups. Statistical analysis revealed no significant differences between Group 1 and Group 2, suggesting that the season of surgery was not associated with a measurable difference in postoperative corneal haze formation or densitometry outcomes in patients treated with MMC-assisted PRK. These findings should not be interpreted as contradicting the previously reported biological role of UV exposure in corneal wound healing or haze formation. Rather, they suggest that, under real-life clinical conditions, undergoing MMC-assisted PRK during months with higher ambient UV radiation did not translate into a measurable increase in postoperative haze or corneal densitometry values. Several factors may explain this difference from previous reports. First, as is routine after refractive laser procedures, all patients were advised to avoid unnecessary direct sunlight exposure and to use UV-protective sunglasses when outdoors during the early postoperative period. Second, ambient environmental UV radiation does not necessarily correspond to actual ocular UV exposure as individual behavior, indoor working conditions, avoidance of direct sunlight, blinking, squinting, and seeking shade may substantially reduce the amount of UV radiation reaching the cornea. In everyday life, patients are unlikely to maintain direct ocular exposure to intense sunlight for prolonged periods. Therefore, even during seasons with higher outdoor UV radiation, the effective UV dose reaching the postoperative cornea may remain limited. Finally, the inclusion of patients with predominantly low-to-moderate myopia and the routine use of intraoperative MMC may also have contributed to the absence of clinically detectable haze in this cohort.

This study has several limitations that should be considered when interpreting the findings. First, its retrospective design introduces inherent methodological constraints. Second, the study population consisted predominantly of patients with low-to-moderate myopia and relatively limited ablation depths. Because higher refractive corrections and deeper stromal ablations are more strongly associated with postoperative haze formation, our findings should not be generalized to all PRK populations, particularly patients undergoing PRK for high myopia. Further studies including eyes with higher refractive errors and greater ablation depths are needed to confirm whether seasonal timing remains unrelated to haze formation in higher-risk PRK cohorts. Another important limitation is that postoperative UV exposure was not measured directly. Seasonal grouping was used as an indirect surrogate for environmental UV intensity, and daily meteorological UV measurements, cloud cover, and sunshine duration corresponding to the exact dates of surgery were not incorporated into the analysis. Therefore, seasonal assignment may not fully capture short-term weather-related fluctuations in actual ambient UV exposure. Moreover, individual variations in ocular UV exposure, including sunglasses use, sunlight avoidance, indoor working conditions, and other protective behaviors, may have influenced the results. Because individual compliance with postoperative UV-protection recommendations and actual UV exposure were not objectively recorded, patient behavior remains a potential confounding factor. The absence of a control group undergoing PRK without MMC is another limitation. Accordingly, although this study evaluates seasonal differences within an MMC-assisted PRK cohort, it cannot determine whether the absence of clinically detectable haze was primarily attributable to MMC or to other factors such as postoperative behavior, refractive range, or individual UV-protective measures. Nevertheless, postoperative sunlight avoidance and UV-protective sunglasses use are routinely recommended after refractive laser procedures. Therefore, the present study reflects real-life clinical practice rather than an unrestricted UV-exposure model. In this context, evaluating patients without excluding their routine daily behaviors during periods of potentially higher UV exposure strengthens the clinical relevance of the findings.

## 5. Conclusions

In this retrospective cohort of patients undergoing MMC-assisted PRK for low-to-moderate myopia, surgical season was not significantly associated with postoperative corneal haze or corneal densitometry outcomes. Rather than directly measuring individual UV exposure, this study reflects real-life clinical outcomes among patients who received standard postoperative UV-protection recommendations. Within this context, the findings suggest that seasonal deferral during high-UV summer months may not be necessary for similar low-to-moderate myopic patients. Further prospective studies including patients with higher refractive errors, deeper ablation depths, and objective UV-exposure assessment are needed to determine whether these findings remain applicable in higher-risk PRK populations.

## Figures and Tables

**Table 1:** Preoperative and Postoperative Clinical Findings Following PRK + MMC Treatment.

Parameter	Preoperative (Mean ± SD)	Postoperative (Mean ± SD)	*p*-Value
Spherical Diopter (D)	−2.30 ± 1.20	−0.26 ± 0.26	<0.001
Astigmatism	−0.86 ± 0.91	−0.19 ± 0.22	<0.001
Spherical Equivalent (SE)	−2.78 ± 1.11	−0.41 ± 0.33	<0.001
Best Corrected Visual Acuity (BCVA)	0.98 ± 0.06	0.98 ± 0.06	—

**Table 2:** Comparison of Corneal Densitometry (CD) Values in Different Corneal Zones Before and After PRK + MMC Treatment.

Parameter	Preoperative (Mean ± SD)	Postoperative (Mean ± SD)	*p*-Value
CD02 (0–2 mm zone)	17.09 ± 1.73	13.40 ± 1.25	<0.001
CD26 (2–6 mm zone)	15.34 ± 1.51	12.28 ± 1.06	<0.001

**Table 3:** Seasonal Group Comparisons and Preoperative-to-Postoperative Changes in CD02 and CD26.

Seasonal Comparison	Group	Preoperative	Postoperative	Preop-Postop Change
CD02 (*p* = 0.168)	Winter (*n* = 42)	16.95 ± 0.35	13.65 ± 0.22	*p* < 0.001
	Summer (*n* = 58)	17.18 ± 0.12	13.21 ± 0.12	*p* < 0.001
CD26 (*p* = 0.284)	Winter (*n* = 42)	15.25 ± 0.29	12.39 ± 0.17	*p* < 0.001
	Summer (*n* = 58)	15.44 ± 0.12	12.15 ± 0.10	*p* < 0.001

## Data Availability

The datasets used in this study are not publicly available due to patient privacy; however, they can be shared upon reasonable request, provided that approval is obtained from the Institutional Ethics Committee.
